# Ex-vivo evaluation of the mucoadhesive properties of *Cedrela odorata* and *Khaya senegalensis* gums with possible applications for veterinary vaccine delivery

**DOI:** 10.1186/s40064-016-2948-0

**Published:** 2016-08-08

**Authors:** Benjamin O. Emikpe, Victor O. Oyebanji, Michael A. Odeniyi, Adebayo M. Salaam, Omolade A. Oladele, Theophilus A. Jarikre, Oluwole A. Akinboade

**Affiliations:** 1 Department of Veterinary Pathology, Faculty of Veterinary Medicine, University of Ibadan, Ibadan, Nigeria; 2 Center for Control and Prevention of Zoonosis (CCPZ), Faculty of Veterinary Medicine, University of Ibadan, Ibadan, Nigeria; 3 Department of Pharmaceutics and Industrial Pharmacy, Faculty of Pharmacy, University of Ibadan, Ibadan, Nigeria; 4 Centre for Drug Discovery, Development and Production, University of Ibadan, Ibadan, Nigeria; 5 Department of Botany, Faculty of Science, University of Ibadan, Ibadan, Nigeria; 6 Department of Veterinary Medicine, Faculty of Veterinary Medicine, University of Ibadan, Ibadan, Nigeria; 7 Department of Veterinary Microbiology and Parasitology, Faculty of Veterinary Medicine, University of Ibadan, Ibadan, Nigeria

**Keywords:** *Cedrela odorata*, Gum polymer, *Khaya senegalensis*, Mucoadhesives, Vaccine, Veterinary

## Abstract

**Background:**

Few studies have investigated the interaction of bioadhesives with biologic tissues for veterinary application. Hence, this study evaluates the mucoadhesive property and vaccine delivery properties of polymers from phytogenic origin. Gums from *Cedrela odorata* and *Khaya senegalensis* were harvested, purified, dried and compressed into 500 mg tablets individually and in combined ratios. The time taken for these tablets, placed on freshly excised (5 × 5 cm) trachea and duodenal tissues of cattle, chicken, pig, sheep and goat and fastened to the basket end of a tablet dissolution machine probe set at 50 rev/min in a phosphate buffer 6.8 pH at 37 °C, to fall off the tissue was the peak adhesion time (PAT). Gum with best PAT was combined with Newcastle disease vaccine and the procedure repeated. Haemagglutination assay (HA) was conducted on the gum polymer-vaccine mix with gum and vaccine individually as controls.

**Results:**

On intestinal and trachea tissues, Cedrela gum polymer averagely had prolonged PAT (≈1 h 30 min and 1 h respectively) while average PAT values of Khaya gums followed the same trend but too transient PAT (≈6 and 0.3 min respectively). However on combination, Cedrela–Khaya polymer mix (1:1) was best on chicken, cattle and sheep trachea and intestinal tissues (PAT of 1 h 30 min and 2 h 24 min respectively). On combination with vaccine, the PAT of the gums reduced slightly on cattle and sheep tissues while other animal tissue showed varied results. The HA results showed the gum polymer boosted the HA property of the vaccine (Log 10^5^), when compared to vaccine alone (Log 10^4^).

**Conclusion:**

Hence, mucoadhesives from phytogenic sources have potential for non-invasive vaccine application.

## Background

Over the past few decades, improving delivery system to optimize drug and vaccine responses has become a research focus. As extensive research is being carried out on discoveries of new drugs so also studies to advance delivery system especially along the non-invasive routes- *the mucosal route* are being conducted (Sumanjali and Sellappan 2013). This was reflected with the global revenue for advanced drug delivery system estimated to be $181.9 billion in 2013 and projected to be about $212.8 billion in 2018 at 3.2 % annual growth (PRNewswire, Sept. 2, 2014).

The mucosal route is the most extensive and first line of barrier most pathogens penetrates in other to establish an infection (Saroj and Bala 2010). It is also the most accessible and non-invasive route for drug and vaccine delivery. Studies also showed that generating protective mucosal antibodies through parenteral vaccination is difficult while obtaining protective mucosal as well as parenteral immunity by inoculating antigen by the mucosal route is often possible (Bye et al. 1984). This gives an edge to the mucosal route over invasive parenteral procedures. Hence the adaptive development of strong immunoprotective mechanisms such as extensive continuous antigenic sampling and induction, immune exclusion and cross protection across mucosal surfaces has made enhancement of these mechanisms of interest to researchers.

However, unpredictable absorptive or antigenic response along this route due to dynamic interplay of physical and chemical mechanisms of homeostatic balance has made this route less preferable to other routes. This was evident in the reports of Sabri et al. (2013), where protective immune response was achieved with the use of intranasal recombinant *Mannheimia hemolytica* vaccine against caprine pneumonia in boer goats.

Hence in view of the potential inherent in the exploration of mucosal immunity, studies aimed at enhancing xenobiotic absorption and modulation of antigenic responses to vaccines along this route is needed. This could be achieved through development and exploration of bioadhesives polymers to enhance bioadhesion to mucosal surfaces such as skin, nasal, buccal, vaginal, and rectal i.e. mucoadhesion which is term used to define the interfacial force interactions between polymeric materials and mucosal tissues (Odeniyi et al. 2015).

When compared to mineral oil or alum-compound widely employed as components of most parenteral vaccines, mucoadhesives could adsorb temporarily to mucus membranes thus decreasing the transit time, potentially forms a temporary depot system for gradual continuous antigen release, activation and recruitment of antigen presenting cells (APCs) with subsequent formation of high affinity antibodies (Khurana et al. 2010; Mohan et al. 2013).

Mucoadhesion mainly occurs in three contiguous stages which begins by an intimate contact between the mucoadhesive polymer and the mucus layer, polymer macromolecular penetration of the mucus layer and ultimately molecular interaction by secondary non-covalent bonds. These bonds have being reported to occur through physical or mechanical interaction through the entanglement of the adhesive material and extensive mucus chains as well as secondary chemical interactions. The latter may be due to electrostatic or hydrophobic interactions, hydrogen or covalent bonding as well as dispersion forces. Several theories have been proposed to explain these fundamental mechanism(s) of attachment. Examples of such are wetting, electronic, adsorption, and diffusion and fracture theories (Dominique and Gilles 1997; Globodanka and Duncan 1997).

Though the application of mucoadhesive polymers from natural sources dates back to the mid-19^th^ century when gum tragacanth and dental adhesive powders were combined to form a delivery vehicle for penicillin application, synthetic polymers with risks of mucosal irritation have been developed and widely explored (Adriaens et al. 2003; Ameye et al. 2005). Examples of plants from which mucoadhesive products have been explored includes locust bean, Xanthum and Okra for controlled release (Xiaohong et al. 2003; Kalu et al. 2007; Beneke et al. 2009; Rohit et al. 2014), karaya, acacia, and cashew gums as suspending, emulsifying and dental adhesives (Munday and Philip 2000; Ahmed and Al-Ghazawi 2005; Shefter 2009), Khaya gum as binding agents (Odeku and Itiola 2003).

Marine sea weed gums, alginates and carrageenan are also examples adhesive polymers from marine while other non-phytogenic polymers explored include Tuftsin, an immunostimulatory peptide; chitosan, chitin, hyaluronic acid and chondroitin sulphate (Rohit et al. 2014; Gao et al. 2015).

Although plants presents with abundant reservoir of these natural polymers known as gums and mucilages which had gained extensive applications such as in food and drug preparations, the usefulness of these polymers in veterinary medicine have not been fully researched. Few studies have elucidated on the potential of Cedrela and Khaya gums as a candidate for drug delivery (Odeniyi et al. 2013, 2015) and as a possible binder in drug preparations respectively. They have neither been individually or in combination explored with respect to veterinary vaccine delivery. The aim of the present preliminary study is to evaluate ex vivo, the interaction of these gum gels with biologic tissues with respect to possible application for non-invasive methods of vaccine delivery in veterinary subjects.

Diverse techniques with different approaches have being proposed for both ex vivo and in vivo evaluation of bioadhesive strength of most polymeric substances. Most ex vivo techniques such as the Wilhelmy plate technique, Electromagnetic force transducer have been used to evaluate tensile stress while adhesion tests based on measurement of the force required to separate two polymer coated glass slides with a film of mucus sandwiched between have been used to evaluate shear stress. Such was the design of a flow chamber method by Mikos and Peppas (1990). Other approaches such as the rheological method (Prabhu et al. 2010; Riley et al. 2001) and the viscometric method (Hassan and Gallo 1990) have also been used to evaluate in vivo behavior of mucoadhesive polymers and quantify mucin-polymer bioadhesive strength respectively. However, in this study, due to limited resources, a tablet dissolution machine was adaptively used to evaluate the interaction between the gum polymer and mucosal tissue surfaces. This attempts to simulate the interplay of factors which plays out between mucosal surface and the mucoadhesive polymer under an in vivo condition.

## Methods

### Quantification of mucin-polymer bioadhesive strength

**Fig. 1:**
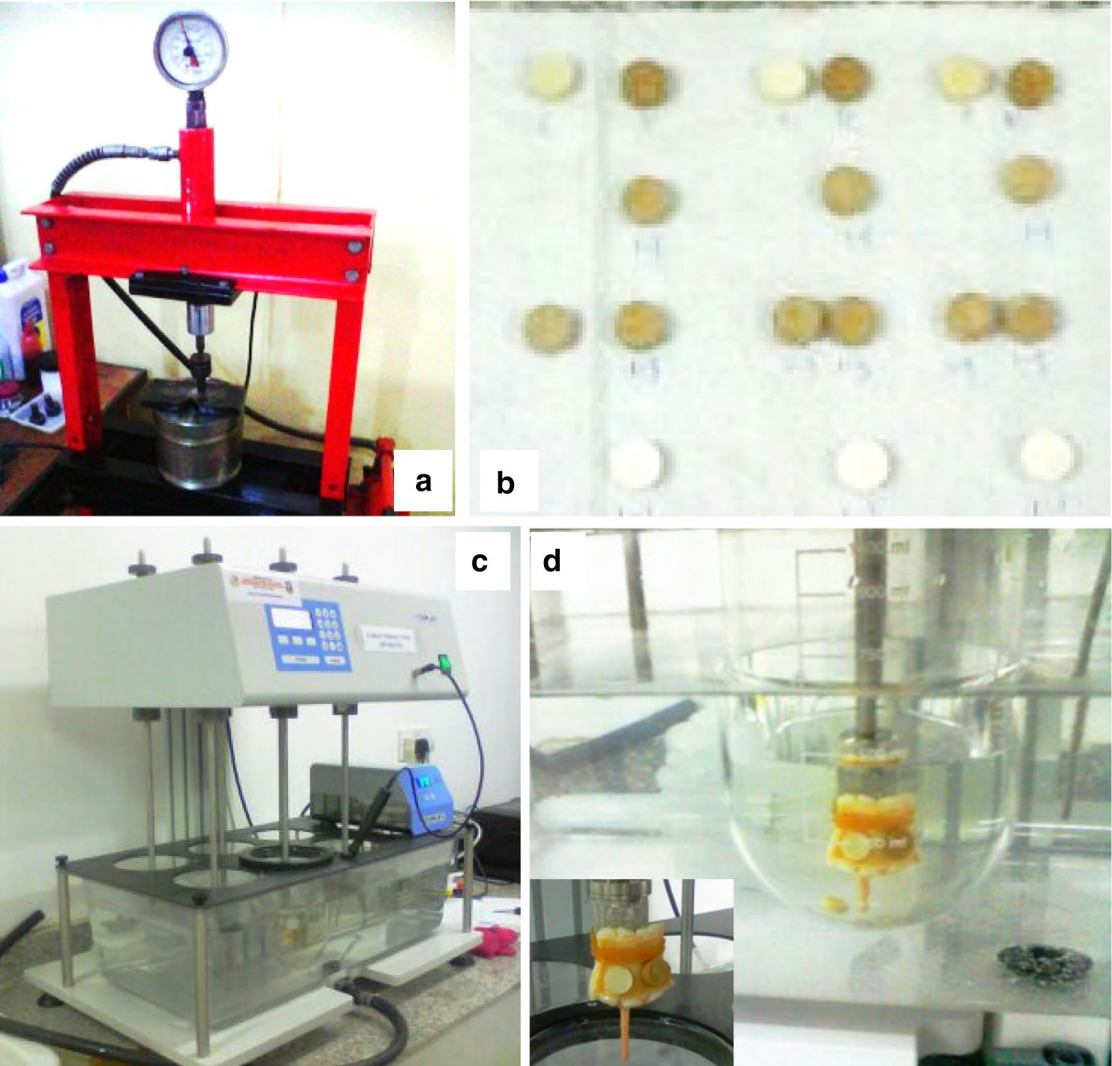
**a**–**d** for Ex-vivo evaluation of the mucoadhesive properties of *Cedrela odorata* and *Khaya senegalensis* gums with possible applications for veterinary vaccine delivery. **a** Hydraulic tableting machine. **b** Tablets of different polymer ratios. **c** Tablet dissolution machine. **d** Tablet on tissue (*inset*) Immersed in phosphate buffer and subsequently in water bath at 37 °C

A tablet dissolution machine (Copley dissolution apparatus) (Fig. 1c) was adaptively used to quantify the mucin-polymer bioadhesive strength. This is a modification to the shear stress measurement technique (Prabhu et al. 2010).

This machine was used to quantify the mucin-polymer adhesive strength by determining the peak adhesion time in a hydrated environment (physiologic buffer, pH and temperature) while the revolution of the machine probe provided a measure of the shear stress of the gum on the biological tissue (Odeniyi et al. 2013).

### Determination of peak adhesion time

The peak adhesion time was the time it takes for a mucoadhesive material compressed into tablet to detach from an animal tissue mounted on the probe of the dissolution machine under physiological conditions which simulates the interplay of in vivo condition.

### Gum preparation

Cedrela and Khaya gums were extracted from incised stems of *Cedrela odorata* and *Khaya senegalensis* trees respectively. The gums were validated to be from *Cedrela odorata* and *Khaya senegalensis* (Desv.) *A. Juss* tress at the Department of Botany Herbarium, University of Ibadan with reference numbers UIH-22378 and UIH-22415, respectively. They were purified at the Department of Pharmaceutics and Industrial Pharmacy, Faculty of Pharmacy, University of Ibadan, using methods described by Odeniyi et al. (2015). Briefly, the exudates were hydrated in 0.5:95.5 (v/v) CHCl_3_-water mixtures for 5 days with intermittent stirring; extraneous materials were removed by straining through a muslin cloth. The gums were precipitated from solution with absolute ethanol which were then filtered, washed with diethyl ether, and dried in hot air oven at 40 °C for 18 h. The gum precipitates were pulverized using a laboratory blender and sieved. 500 mg of each gum were measured and compressed into tablets (Fig. 1b) at a pressure of 1 kg using a Hydraulic tableting machine (Fig. 1a).

### Tissue preparation

About 5 cm × 5 cm each of freshly excised trachea and duodenal tissues from five domestic animals namely *Cattle, Chicken, Pig, Goat* and *Sheep* were collected from the abattoir and used for the experiment.

### Experimental procedure

The freshly excised and trimmed animal tissues were mounted on the basket end attached to the probe of the Tablet dissolution machine (Fig. 1c). The tissues were fastened to the basket end with an elastic band and the gum tablet placed on the mucosal surface carefully. This was then lowered into a beaker containing phosphate buffer solution of pH 6.8. The beaker sits within a large water bath with temperature set at 37 °C. The machine was then set at 50 revolutions per minute which mimics the in vivo physiologic fluid, pH, temperature and peristaltic movement of contractile smooth muscles.

The time it took for a gum tablet to fall off the tissue is recorded as the peak adhesion time (PAT) (Fig. 1d). This adhesion time is a reflection of the strength of interaction of the gum molecules with the mucosa layer of the tissues under simulated in vivo conditions. This procedure was conducted in triplicates on the same tissues from different domestic animals as stated above. The average peak adhesion time with standard deviation was evaluated using Microsoft Excel software package.

### Haemagglutination assay

The gum with the best PAT, was then combined with lyophilized Newcastle disease vaccine (Indovax; dosage of 200 birds containing ≥10^6^ ED_50_) in the ratio 2:2:1 (Cedrela:Khaya:Vaccine) under cold chain, processed into tablets and the procedure above repeated for all domestic animal tissues stated earlier.

Haemagglutination assay was also carried out at the Avian Research Unit, Department of Veterinary Medicine, and University of Ibadan on the gum polymer-vaccine mix with gum alone and vaccine alone as controls. Protocols from the Laboratory were also employed for the test. Briefly, the gum polymer vaccine mix was dissolved in 8 ml of normal saline; 50 μl of normal saline was dispensed into each microtitre well while 50 μl of the antigenic solution (gum-vaccine mix) was added into the first well in each row. With the micropipette, the content of the first row was mixed and 50 μl transferred into the second row of well. The procedure was repeated serially for all rows of well with the last 50 μl dispensed off. 50 μl of 0.5 % chicken red blood cell was then added to each well, mixed briefly together on a micro-shaker (Flow Laboratories, GmbH Diezstrabe 10, 5300, Bonn 3**)** and left on the bench for 30 min. The last dilution which shows Haemagglutination was taken as the titer of the antigen.

Furthermore, checkerboard dilution was also carried out on gum polymer mix sample that gave the best PAT. This dilution were then split in two sets, a part was heat inactivated in water bath at 56 °C for 30 min while the other part was left on the bench. HA assay was then repeated for the gum polymer mix alone to ascertain the lowest dilution with the least agglutinating property.

## Results

The peak adhesion time (PAT) for *Cedrela odorata* and *Khaya senegalensis* gum tablets are given in Table 1.

**Table 1:** Peak adhesion time for Cedrela gum polymer

	Cedrela gum average ± SD (mins)	Khaya gum average ± SD (mins)
Cattle		
Trachea	94.3 ± 23.6	0.55 ± 0.05
Intestine	117.8 ± 39.8	4.63 ± 1.91
Chicken		
Trachea	111.1 ± 15.6	0.20 ± 0.10
Intestine	55.5 ± 33.0	10.07 ± 2.93
Pig		
Trachea	51.1 ± 20.0	0.07 ± 0.05
Intestine	76.7 ± 11.5	8.33 ± 2.32
Goat		
Trachea	8.2 ± 6.0	0.20 ± 0.00
Intestine	106.7 ± 15.3	3.10 ± 0.87
Sheep		
Trachea	22.6 ± 7.9	0.17 ± 0.06
Intestine	131.7 ± 72.5	3.10 ± 0.87

From Table 1, Cedrela gum was strongest on the intestine of all animal species except the chicken. Hence from the above, Cedrela gum will be best suitable as a vehicle for vaccines or drugs delivered along the gut mucosa in most domestic animals except in the chicken where it appears to be more adaptable along the airway mucosal e.g. spray vaccines. The PAT of Khaya gum was also strongest on intestine and therefore would be best adapted as a delivery vehicle for gut vaccines and drugs than for the airway. However, when compared with Cedrela gum, Khaya gum adhesion action time appears too transient while that of Cedrela appears moderate. Therefore a combination of both gums at varying ratios was done to determine the effect of Cedrela gum polymer on Khaya gum polymer adhesion time.

Both gum polymers were combined in ratios 1:1, 1:3 compressed at the same pressure as was the individual polymers and PAT evaluated. The results obtained are given in Table 2.

**Table 2:** Peak adhesion time for Cedrela-Khaya gum polymer mix in ratios 1:1, 1:3

	Ced: Khy 1:1 average ± SD (mins)	Ced:Khy 1:3 average ± SD (mins)
Cattle		
Trachea	185.00 ± 35.00	35.53 ± 7.82
Intestine	246.67 ± 84.10	180.33 ± 17.90
Chicken		
Trachea	58.30 ± 19.26	9.37 ± 6.44
Intestine	156.10 ± 17.66	167.1 ± 23.92
Pig		
Trachea	21.00 ± 14.30	11.33 ± 6.55
Intestine	156.10 ± 17.67	94.80 ± 16.63
Goat		
Trachea	22.33 ± 6.97	24.87 ± 2.48
Intestine	118.3 ± 67.08	93.53 ± 72.78
Sheep		
Trachea	168.27 ± 133.78	10.83 ± 5.22
Intestine	156.77 ± 23.69	162.00 ± 17.44

From Table 2 PAT of Cedrela-Khaya 1:1 gum polymer mix increased considerably on all trachea tissues except pig and chicken when compared to either gum polymer singly. Also a considerable increase was observed on the intestine mucosa from all the animals used compared to both gums singly. Hence, the use of both gum combined in equal ratio as a delivery vehicle would be best preferred for oral and spray based vaccines and drugs as opposed to the gums singly except with few exceptions. While the 1:3 gum polymer mix improved on trachea tissues but not as 1:1 gum polymer mix on gut mucosa which makes it a potential candidate for drugs and vaccines along that route. However, on the trachea mucosa, the adhesion force was weak when compared to the 50 % each combination ratio or Cedrela gum alone but better than Khaya alone

Overall the gum polymers and their combinations adhesion action in each animal tissue from highest to the least are given as follows in Table 3.

**Table 3:** Summary of Peak adhesion time of phytogenic polymer used from highest to lowest on trachea and intestines of domestic animals

S/no	Animal	Tissue	Highest → lowest			
1	Cattle	Trachea	Ced: Kha1:3	Ced: Kha1:1	Ced. 100 %	Khaya 100 %
		Intestine	Ced: Kha 1:1	Ced: Kha 1:3	Ced. 100 %	Khaya 100 %
2	Chicken	Trachea	Ced. 100 %	Ced: Kha 1:1	Ced: Kha 1:3	Khaya 100 %
		Intestine	Ced: Kha 1:3	Ced: Kha 1:1	Ced. 100 %	Khaya 100 %
3	Pig	Trachea	Ced. 100 %	Ced: Kha 1:1	Ced: Kha 1:3	Khaya 100 %
		Intestine	Ced: Kha 1:1	Ced: Kha 1:3	Ced. 100 %	Khaya 100 %
4	Goat	Trachea	Ced: Kha 1:3	Ced: Kha 1:1	Ced. 100 %	Khaya 100 %
		Intestine	Ced. 100 %	Ced: Kha 1:3	Ced: Kha 1:1	Khaya 100 %
5	Sheep	Trachea	Ced: Kha 1:1	Ced. 100 %	Ced: Kha 1:3	Khaya 100 %
		Intestine	Ced: Kha 1:3	Ced: Kha 1:1	Ced. 100 %	Khaya 100 %

On all trachea tissues, the peak adhesion strength was divided between Cedrela Khaya 1:3 gum polymer mix, Cedrela alone and Cedrela Khaya 1:1 gum polymer mix. While on all intestinal tissues, the peak adhesion strength was divided between Cedrela Khaya 1:3, Cedrela Khaya 1:1 combinations and Cedrela alone. Overall however, on cattle, chicken and sheep tissues, Cedrela-Khaya (1:1) mix had highest PAT; on goat tissues, Cedrela-Khaya (1:3) mix while either Cedrela-Khaya (1:1) mix or Cedrela alone would be adaptively reliable for pig tissues.

On combination with vaccine, the PAT of the gums reduced slightly on cattle and sheep tissues while other animal tissue showed varied results. Although the vaccine used was primarily a poultry vaccine and the result obtained on tissues of other species might not be reflection of what could be obtained with specie specific vaccines, it however gave a rough idea of the interaction of the gum polymer mix with vaccines on mucosa surfaces especially with regards to poultry species.

Haemagglutination assay results showed the gum polymer boosted the HA property of the vaccine (Log 10^5^), when compared to vaccine alone (Log 10^4^). Although the gum polymer mix alone also showed HA property (Log 10^5^) equivalent to the gum polymer mix and vaccine combination (Table 4).

**Table 4:** Gum polymer mix and vaccine combination haemagglutination assay results

S/no	Test	Antigenic titer (Log_2_)
1.	Vaccine	4
2.	Vaccine + Gum (Ced:Khy) Combination	5
3.	Gum (Ced + Khy) alone	5
4.	Gum (Cedrela)	5
5.	Gum (Khaya)	5

Due to hemagglutinating property, the results of the checkerboard dilution conducted on both bench and heat inactivated gum polymer sets are presented below in Table 5.

**Table 5:** Gum polymer mix, checkerboard dilution and haemagglutination assay results

S/no	Test dilution	Antigenic titer (bench) (Log_2_)	Antigenic titer (heat inactivated)
1.	Gum mix (neat)	5	4
2.	Gum mix (1:2 dilution)	4	4
3.	Gum mix (1:4 dilution)	3	4
4.	Gum mix (1:8 dilution)	2	2
5.	Gum (1:16 dilution)	2	2

From Table 5 above, the heat inactivated gum mix had lower heamagglutinating property than the bench at both neat and 1:2 dilutions. However, at 1:4, 1:6, 1:8 dilutions, the titer was the same. Hence 1:8 bench dilution with the least agglutinating titer was recommended as a delivery vaccine vehicle in an in vivo model study.

## Discussion

Current research in vaccine development focuses on development of vaccine protocols requiring single or multiple non-invasive administration, since the major disadvantage of many currently available vaccines is that most are applied through parenteral routes and repeated administrations are required (Saroj and Bala 2010) Repeated vaccine application is especially needful in Nigeria where Newcastle disease (ND), a per acute, acute and sometimes subclinical contagious disease of poultry is endemic (Health et al. 1999; Oladele et al. 2002). Mortality from frequent outbreaks often reaches 100 % (Alders and Spradbrow 2001).

The main routes of entry of many poultry viral diseases including Newcastle disease which affect respiratory, gastrointestinal and nervous systems are that of mucosal route (respiratory through inhalation, intestinal through ingestion) (Whiteman and Bickford 1983). Hence a potent means of defense might be by improving antigenic induction with subsequent improved effector mechanisms along these routes. This pattern was verified in the reports of Emikpe et al. (2013) where pathological evaluation of goats vaccinated intranasally showed no pneumonic lung lesion compared to the observed lesions in the subcutaneously, intramuscularly vaccinated groups. Ezeasor et al. (2013, 2014) also reported improved lymphoproliferative responses when haematological changes associated with intranasal and parenteral routes of vaccination against PPR virus was evaluated in West African dwarf goats.

In poultry, although vaccine delivery along these mucosal routes are already in use, but limiting factors such as unpredictable or limited immune responses, shortened protection interval, endemicity of the disease leading to frequent outbreaks have resulted in disharmonized vaccine protocol among farmers (Okwor et al. 2010; Oluwole et al. 2012). These reasons coupled with search for non-invasive methods of vaccine administration which could evoke better protective responses led to the development and standardization of mucoadhesives as vehicle for vaccine delivery.

From this study, going by the interaction of mucoadhesives (gum gels) from the phytogenic sources used, there is a rapid and prolonged adherence to the mucosa surface. This was observed in the gum polymer combination which could be attributed to a possible synergistic effect of the phytogenic mucoadhesives on the tissues.

Also, the viscoelastic properties exhibited by the phytogenic mucoadhesives in hydrated environment was good as revealed by the strong interaction with mucin on the mucosal tissue with minimal variation and breakdown at the mucoadhesive mucus interface of adhesion. This showed a good macromolecular interpenetration effect to form a stable hydrated gel by bonds (such as weak van der Waals and hydrogen bonding) and secondary interactions (glycoproteins). The breakdown observed at the mucus layer as opposed to the mucoadhesive-mucus interface plane of adhesion showed that mucoadhesives from these phytogenic sources are strong enough to cause a prolonged and stable interaction with the mucus layer of mucosal surfaces. Thus facilitating a prolonged time for continuous antigenic presentation, stimulation and response (Parthasarathy et al. 2011) and would be well adaptable for an in vivo study.

Further, the interference by hydrodynamic conditions, pH and vaccine components were moderate, especially for the latter. This places the mucoadhesive as a good candidate for vaccine vehicle especially in veterinary subjects as it showed good inter-penetration with vaccine antigens, retention of mucoadhesive property and boost in Haemagglutination property of the gum polymer-vaccine mix. This latter activity is a desirable factor in the design of an ideal adjuvant which can possibly provide an immunopotentiating response to vaccines under in vivo conditions. Also, the observed haemagglutination activity of these mucoadhesives could be due to the presence of lectins which is reported to be abundant in plants and its products. These ubiquitous highly specific sugar-binding proteins also have mitogenic, antibacterial, antifungal, antiviral as well as cytotoxic activities (Ingale and Hivrale 2013).

Therefore mucoadhesives from phytogenic origin shows potential as bioadhesive delivery vehicle for vaccines in veterinary subjects, however, these claims need to be validated under in vivo conditions.
